# Improving phase II oncology trials using best observed RECIST response as an endpoint by modelling continuous tumour measurements

**DOI:** 10.1002/sim.7453

**Published:** 2017-08-28

**Authors:** Chien‐Ju Lin, James M.S. Wason

**Affiliations:** ^1^ MRC Biostatistics Unit University of Cambridge U.K.; ^2^ Institute of Health and Society Newcastle University U.K.

**Keywords:** continuous tumour shrinkage endpoints, longitudinal model, phase II cancer trial

## Abstract

In many phase II trials in solid tumours, patients are assessed using endpoints based on the Response Evaluation Criteria in Solid Tumours (RECIST) scale. Often, analyses are based on the response rate. This is the proportion of patients who have an observed tumour shrinkage above a predefined level and no new tumour lesions. The augmented binary method has been proposed to improve the precision of the estimator of the response rate. The method involves modelling the tumour shrinkage to avoid dichotomising it. However, in many trials the best observed response is used as the primary outcome. In such trials, patients are followed until progression, and their best observed RECIST outcome is used as the primary endpoint. In this paper, we propose a method that extends the augmented binary method so that it can be used when the outcome is best observed response. We show through simulated data and data from a real phase II cancer trial that this method improves power in both single‐arm and randomised trials. The average gain in power compared to the traditional analysis is equivalent to approximately a 35% increase in sample size. A modified version of the method is proposed to reduce the computational effort required. We show this modified method maintains much of the efficiency advantages.

## 1 INTRODUCTION

A new cancer treatment is tested for potential benefit in phase II trials that use a relatively small number of patients followed over a short period of time. The results of the phase II trial determines whether to test the treatment in a larger, more time‐consuming, and more costly phase III trial. Because of the high cost of, and high failure rate in, phase III oncology trials,^1^ it is important to improve the analysis of phase II trials to ensure the decision is more accurate.

Phase II oncology trials use a variety of endpoints to evaluate the efficacy of a treatment.(2, 3) The most commonly used endpoints in solid tumours are based on the Response Evaluation Criteria in Solid Tumours (RECIST) scale.^4^ The RECIST defines tumour size as the sum of longest diameters of target lesions and categorises patients into complete response (CR), partial response (PR), stable disease (SD), and progressive disease (PD). The CR and PR represent no new tumour lesions and a 100% shrinkage and greater than 30% shrinkage, respectively; PD represents a 20% increase in tumour size from the minimum size observed up to that point or new lesions appearing. Often patients are followed until they are categorised as PD or a preplanned time, and patients with CR or PR are labelled responders. The endpoints for evaluating a treatment include response rate and time to an event (progression/death). The response rate is defined as either (1) proportion of patients who are responders at a certain time after baseline (fixed response) or (2) the proportion of patients whose best observed response (BOR) before progression is CR or PR. A response endpoint classing CR and PR as success is called objective response rate (ORR), which is often used in practice. Disease control rate additionally includes SD as success. In some cases where response rate may not be the optimal method, a time to event endpoint “progression‐free survival” is considered, which is the time until PD or death. Response‐based endpoints are an indicator of the relative anti‐tumour activity of the treatments and are not always highly predictive of overall survival. Improving the efficiency of trials using response‐based endpoints would help increase the success rate of phase III trials in tumour types where response is predictive of overall survival.

Categorizing patients into responders and nonresponders is widespread and clinically appealing. However, it can have substantial statistical disadvantages. Its major limitation is that it dichotomises the continuous tumour variable, thus discarding information. This loses substantial efficiency.^5^ Some researchers have addressed the problem and proposed methods to make use of the continuous change in tumour response to improve statistical efficiency. This is done in different ways. Karrison et al^6^ propose directly using the change in tumour size as an endpoint. Wason and Seaman^7^ use models for the tumour size and new lesion data which can be used to infer the fixed response rate with higher precision. They find the Karrison's method was more powerful when the probability of new lesions was different between arms but less powerful when the mean tumour size change was different. Jaki et al^8^ propose a method that links tumour size change with mortality using historical datasets. Authors have demonstrated that using continuous scales can increase the power (or reduce the required sample for a target power) compared to analysing the binary composite outcome.

Focusing on response‐based endpoints, the method of Wason and Seaman^7^ retains the clinically meaningful endpoint but takes into account the continuous information on tumour size. This method is limited by only allowing 2 follow‐up visits and only considering response rate at a fixed time (ie, it cannot be used to make inferences on BOR). In trials in which patients are assessed twice (interim and final), their method is sufficient. However, in trials where patients are followed up until a preplanned time, a method incorporating information on all measurement data is preferred for efficiency reasons. In this paper, we consider ORR and present an extended method that can be used for any number of follow‐up times for fixed response or BOR. We propose a modified version that uses a highly efficient technique for multivariate integration,^9^ which substantially reduces the computation time taken. We assess the properties of the proposed methods by using simulated data and data from a real phase II cancer trial (HORIZON II).

This paper is divided into 4 sections. Section 2 gives a brief overview of the augmented binary method (Augbin).^7^ It then describes the proposed extensions of the method. Section 3 evaluates the performance of the proposed methods using simulations and real data. Section 4 summarizes the results and presents limitations and future work.

## 2 METHODS

### 2.1 Background

We use the phrase “tumour size” as shorthand for the sum of the longest diameter of target tumour lesions. We assume patients tumour sizes are recorded until progression occurs or until a preplanned number of visits. We note there are 2 ways in which a progression can occur: an increase in tumour size by more than 20% (a tumour‐growth progression) or new lesions appearing (a new‐lesion progression). Two response‐based endpoints can be used in the analysis, one being fixed time and the other being BOR. Analysis at a fixed time t uses the proportion of responders at time t (those who have a tumour size shrinkage at time t above a predefined threshold and no progression up to that point). Best observed response defines patients as a responder or not according to their BOR before progression. The latest RECIST guidelines^4^ give BOR 2 definitions according to whether confirmation is required or not. Confirmation means that an apparent response must be backed up by continued response at the next time point to be counted as genuine. This is especially recommended for single‐arm trials. When confirmation is not required (randomised trials comparing 2 arms), BOR is defined as the best response across all time points up to progression. When confirmation is required, BOR is defined as a response if the patient is a responder at 2 consecutive time points before progression.

### 2.2 Notation

Tumour sizes for each patient are measured at several discrete times (T denoting the maximum time). The tumour size at time t for patient i is denoted by z_it_ where t=0 represents the baseline measurement. We denote G and X as the time at which a tumour‐growth progression and new‐lesion progression occurs, respectively. Once a patient progresses they are no longer followed up. The observed data is therefore (F_i_,**z**_i_) where $F_{i}=\min(X_{i},G_{i},T)$. We define y_it_ as the log tumour size ratio for patient i at time t, $y_{it}=log(z_{it}/z_{i0})$, and c_t_ as the prespecified dichotomisation threshold for response (on the log tumour ratio scale). Further, D_it_ defines new‐lesion progression indicators: {D_it_=1 if patient i has a progression due to new lesions occurring between time (t−1) and t, t=1,…,T }. For simplicity, we define composite indicators S for fixed time and BOR for best observed response using tumour progression relative to the baseline rather than nadir (the lowest rumour size observed so far). Note that we show how to use the true definition in the Supporting Information. In addition, we assume that response at a fixed time would mean just the response status at the specified time is of interest. The response indicator for patient i using fixed time is defined as

$$\begin{array}{c} S_{it}=\left\{\begin{array}{cc} 1, & \;\;\text{if}\;D_{ij}=0\;\text{for all}\;\;j=1,\text{⋯},t,y_{it}<c_{t}\;\text{and no tumour progression before}\;t,\\ 0, & \;\;\text{otherwise}. \end{array}\right. \end{array}$$

For BOR, when confirmation is not required, the event is equivalent to having at least 1 record classified CR/PR before progression or time T, the response indicator B O R_i_ is defined as

$$\begin{array}{c} BOR_{i}=\left\{\begin{array}{cc} 1, & \;\;\text{if there exists a}\;t\;\text{such that}\;y_{it}<log(0.7),t⩽min(F_{i},X_{i}-1)\;\text{and}\;log(0.7)<y_{i1},\text{⋯},y_{i(t-1)}<log(1.2),\\ 0, & \;\;\text{otherwise}. \end{array}\right. \end{array}$$

We consider the case where confirmation is required later. We lay out more fully how to incorporate the actual definition of progression with change from nadir in the Supporting Information. This includes use of an indicator function for the extended augmented binary method (eAugbin) and a more exact method for the modified augmented binary method (mAug).

### 2.3 Estimating response probability using the Augbin with 2 follow‐up times

The augmented binary method, henceforth referred to as Augbin, was proposed by Wason and Seaman.^7^ We briefly describe this method here, but more details are found in Wason and Seaman.^7^

The Augbin method makes assumptions that the log tumour size ratios follow a multivariate normal distribution, and the probability of new‐lesion progression depends only on the observed tumour size at the previous visit. The log tumour size ratios are modelled by

$${\left(Y_{i1},Y_{i2}\right)}^{\prime }|z_{i0}∼N({\left(\mu_{i1},\mu_{i2}\right)}^{\prime },\Sigma ),$$

where μ_i1_=β_1_+ω z_i0_, μ_i2_=β_2_+ω z_i0_. The new‐lesion progression is modelled by using logistic regression models

$$\text{Logit}\{\mathrm{Pr}(D_{i1}=1|z_{i0})\}=\alpha_{1}+\gamma_{1}z_{i0},$$

$$\text{Logit}\{\mathrm{Pr}(D_{i2}=1|D_{i1}=0,z_{i0},z_{i1})\}=\alpha_{2}+\gamma_{2}z_{i1}.$$

The probability of response for patient i at time 2 is written by

$$\mathrm{Pr}(S_{i2}=1|\theta )=\int_{-\infty }^{c_{2}}\int_{-\infty }^{\infty }\mathrm{Pr}(D_{i1}=0|z_{i0})\mathrm{Pr}(D_{i2}=0|D_{1}=0,z_{i0},z_{i1})f_{Y_{1}Y_{2}}(y_{i1}y_{i2};\theta )dy_{i1}dy_{i2},$$

where θ is the vector of parameters from the above models and c_2_ is the dichotomisation threshold (usually log(0.7), representing at least a 30% shrinkage in the tumour size from baseline). The mean response probability is estimated by $\bar{\mathrm{Pr}}(S_{2}=1|\hat{\theta })=\frac{1}{n}\sum_{i=1}^{n}\mathrm{Pr}(S_{i2}=1|\hat{\theta })$, where $\hat{\theta }$ is the maximum likelihood estimator of θ. A program is available in the paper which uses R2Cuba to compute the above integration. An approximately (1‐α)% confidence interval for the probability of response is constructed on the logit scale, that is, expit $\left\{l(\hat{\theta })\pm \Phi^{-1}(1-\frac{\alpha }{2})\sqrt{\mathrm{var}(l(\hat{\theta }))}\right\}$, where $l(\theta )=log\frac{\mathrm{Pr}(S_{2}=1|\theta )}{1-\mathrm{Pr}(S_{2}=1|\theta )}$ and $\mathrm{var}(l(\hat{\theta }))$ is obtained by using the delta method.

### 2.4 Extended Augbin at a fixed time (t>2)

We use the same assumptions and extend the Augbin to t follow‐up times. The log tumour size ratios are modelled by

(1) $${\left(Y_{i1},\text{⋯},Y_{it}\right)}^{\prime }|z_{0}∼N({\left(\mu_{i1},\text{⋯},\mu_{it}\right)}^{\prime },\Sigma ).$$

An unstructured covariance matrix is used (although an alternative form may be needed if t is large enough). The new‐lesion progression is modelled by

(2) $$\text{Logit}\{\mathrm{Pr}(D_{it}=1|D_{i1}=\text{⋯}=D_{i(t-1)}=0,z_{i0},\text{⋯},z_{i(t-1)})\}=\alpha_{t}+\gamma_{t}z_{i(t-1)}.$$

We assume that the new‐lesion progression depends only on the previous observed tumour size. The missing tumour size because of new‐lesion progression can be, therefore, treated as missing at random (MAR) as justified in Wason and Seaman.^7^ $\mathrm{Pr}(Y_{i(t+1)}\;\text{is missing}\;|z_{i0},\text{⋯},z_{iT})=\mathrm{Pr}(Y_{i(t+1)}\;\text{is missing}\;|z_{i0},\text{⋯},z_{it})$. See the “sequential missingness at random” section in Supporting Information for details. We also assume that dropout for other reasons before preplanned time is MAR. The probability of response for patient i at time T can be written by

(3) $$\begin{array}{cc} \mathrm{Pr}(S_{iT}=1|\theta ) & =\int_{-\infty }^{c_{T}}\int_{-\infty }^{c}\text{⋯}\int_{-\infty }^{c}\;\prod_{t=1}^{T}\mathrm{Pr}(D_{it}=0|D_{i1}=\text{⋯}=D_{i(t-1)}=0,z_{i0},\text{⋯},z_{i(t-1)})\\ & \;\times \;f_{Y_{1},\text{⋯},Y_{T}}(y_{i1},\text{⋯},y_{iT};\theta )dy_{i1}...dy_{iT}, \end{array}$$

where c_T_ and c are the dichotomisation cut points (usually log(0.7) and log(1.2), representing at least a 30% shrinkage in the tumour size from baseline and an increase in tumour size by more than 20%). Note that the response at a fixed time $T^{\prime }⩽T$ can be obtained by replacing T by $T^{\prime }$ in the above formulas. The advantage is that the Equation 3 uses the models to estimate probability of response of patients and missing data are MAR, it can be applied to patients who drop out or progress before preplanned time. The probability is interpreted as the probability of patient i being a responder at time T as if they were observed until T. A potential issue of Equation 3 is that the multivariate integration is computationally intensive. The mean response probability is estimated by averaging response probability over n patients given $\hat{\theta }$. An approximately (1−α)% confidence interval is constructed as described in Section 2.3.

### 2.5 Modified Augbin at a fixed time

The objective for this section is to efficiently estimate the mean response probability using continuous tumour‐size information in a computationally efficient way. We assume that {no new‐lesion progression occurs from time 1 to time T} and {no tumour‐growth progression} are conditionally independent given tumour size ${\bar{z}}_{t-1}=(z_{0},\text{⋯},z_{t-1})$. We note this is a strong assumption and assess the sensitivity to this assumption later on. The probability of response for patient i at a fixed time t can be written by

$$\begin{array}{cc} \mathrm{Pr}(\text{response}\;|{\bar{z}}_{t-1}) & =\mathrm{Pr}(\text{no new‐lesion progression until}\;t|{\bar{z}}_{t-1})\\ & \;\times \mathrm{Pr}(\text{no tumour progression at time}\;t|{\bar{z}}_{t-1}). \end{array}$$

Let π_t_ be the probability of new‐lesion progression at time t,t=1,⋯,T. Note that π_t_ is a conditional probability given no new‐lesion progression occurring at previous time points. The log tumour size ratio **Y**_i_ is allowed to depend on baseline tumour size whereas new‐lesion progression depends on the previous observed tumour size at the previous visit. We can model Y by

(4) $${\left(Y_{i1},\text{⋯},Y_{it}\right)}^{\prime }|z_{0}∼f_{Y_{i}}(\cdot ),l(\pi_{it})=\omega_{t}+\beta_{t}z_{i(t-1)}.$$

where f_Y_(·) is a joint distribution and l(·) is the logit link function. We assume that $\pi_{it}({\bar{z}}_{t-1})=\pi_{it}(z_{t-1})$. The probability of response for patient i at a fixed time T can be written by

(5) $$\mathrm{Pr}(S_{iT}=1|{\bar{z}}_{t-1},\theta )=\overset{T}{\underset{t=1}{\prod }}\{1-\pi_{it}({\bar{z}}_{t-1},\theta )\}\int_{-\infty }^{c_{T}}\int_{-\infty }^{c}\text{⋯}\int_{-\infty }^{c}f_{Y_{1},\text{⋯},Y_{T}}(y_{i1},\text{⋯},y_{iT}|z_{i0},\theta )dy_{i1}...dy_{iT},$$

where c_T_ and c are the dichotomisation threshold and θ is a vector of parameters of the models. We assume that f_Y_(·) is the probability density function of a multivariate normal distribution. The multivariate integration can then be calculated by a highly efficient technique proposed by Genz and Bretz.^9^ The observed data for patient i is (F_i_,z_i_). The l(π_i,F+1_) can be estimated by ω_F+1_+β_F+1_ z_i,F_. Their probability of new‐lesion progression at time t,t⩾F_i_+2 is estimated by

(6) $${\tilde{\pi }}_{it}=\frac{1}{n_{t}-k}\sum_{j,y_{jt}\in \varphi }\pi_{jt},$$

where n_t_ is the number of patients with observed z_t−1_ and k is the number of patients who have log tumour size ratio y_t_ outside of the region of integration φ of Equation 5. We trim those k patients to avoid underestimating π_it_. This is similar to an idea of trimmed mean, which is used in many areas and has advantages under both normal and nonnormal distributions.^10^, ^11^

The vector θ consists of (T+1) parameters that make up μ, T(T+1)/2 parameters for Σ, and 2T parameters for the logistic models. The mean response probability is estimated by $\bar{\mathrm{Pr}}(S_{T}=1|\hat{\theta })=\frac{1}{n}\sum_{i=1}^{n}\mathrm{Pr}(S_{iT}=1|\hat{\theta })$, where $\hat{\theta }$ is the maximum likelihood estimator of θ. A (1−α)% confidence interval for $\bar{\mathrm{Pr}}(S_{T}=1|\theta )$ can be constructed:

$$[\bar{\mathrm{Pr}}(S_{T}=1|\hat{\theta })-\Phi^{-1}(1-\alpha /2)\sqrt{\mathrm{Var}(\bar{\mathrm{Pr}}(S_{T}=1|\hat{\theta }))},\bar{\mathrm{Pr}}(S_{T}=1|\hat{\theta })+\Phi^{-1}(1-\alpha /2)\sqrt{\mathrm{Var}(\bar{\mathrm{Pr}}(S_{T}=1|\hat{\theta }))}\;],$$

where Φ is the standard normal distribution function. However, we found that the method has better properties if we find a confidence interval for logit $\left\{\bar{\mathrm{Pr}}(S_{T}=1|\hat{\theta })\right\}$ and transform back. Let $l(\theta )=log\frac{\mathrm{Pr}(S_{T}=1|\theta )}{1-\mathrm{Pr}(S_{T}=1|\theta )}$, we obtain $\mathrm{Var}(l(\hat{\theta }))$ by using the delta method, which is written by

$$\mathrm{var}(l(\hat{\theta }))\approx {\left(\nabla l(\hat{\theta })\right)}^{T}\mathrm{var}(\hat{\theta })\nabla l(\hat{\theta }),$$

where $\nabla l(\hat{\theta })$ is the partial derivatives of l(θ). An approximately (1−α)% confidence interval for the probability of response is

$$[\text{expit}\{l(\hat{\theta })-\Phi^{-1}(1-\alpha /2)\sqrt{\mathrm{var}(l(\hat{\theta }))}\},\text{expit}\{l(\hat{\theta })+\Phi^{-1}(1-\alpha /2)\sqrt{\mathrm{var}(l(\hat{\theta }))}\}].$$

To summarise, the modified method uses a simplification for the relationship between new‐lesion progressions and tumour‐growth progressions to use a more efficient procedure for multivariate integration.

### 2.6 Proposed method for BOR

We focus on the case where confirmation is not required but show briefly how the methodology can straightforwardly allow for confirmation later. By the definition of BOR, a patient is a responder if they have at least 1 log tumour size ratio smaller than log(0.7) before progression or maximum follow‐up time. We define $\Omega_{1}=(log(0.7),log(1.2))$, $\Omega_{2}=(-\infty ,log(0.7))$, and $\Omega_{3}=(-\infty ,\infty )$ as the possible regions of integration corresponding to being classified as stable disease, responder, and irrelevant variables. Let h be the time at which the patient is first classified as CR/PR. Hence, each component of ${\bar{Y}}_{T}$ will fall into 1 of the 3 regions as

(7) $$(Y_{1}\text{⋯}Y_{h-1}\in \Omega_{1},Y_{h}\in \Omega_{2},Y_{h+1}\text{⋯}Y_{T}\in \Omega_{3}).$$

The probability of response using BOR for patient i will be the sum over all possibilities of when the CR/PR is first observed. Following the concept of the eAugbin, the probability of response can be written by

(8) $$\begin{array}{cc} \mathrm{Pr}(BOR_{i}=1|\theta ) & =\sum_{h=1}^{T}\int_{\overset{T-h}{\underset{3}{\Omega }}}\int_{\Omega_{2}^{1}}\int_{\Omega_{1}^{h-1}}\prod_{t=1}^{h}\mathrm{Pr}(D_{it}=0|D_{i1}=\text{⋯}=D_{i(t-1)}=0,z_{i0},\text{⋯},z_{i(t-1)})\\ & \;\times \;f_{Y_{1},\text{⋯},Y_{T}}(y_{i1},\text{⋯},y_{iT};\theta )dy_{i1}...dy_{iT}. \end{array}$$

Similarly, following the concept of the mAug, the probability of response can be written by

(9) $$\mathrm{Pr}(BOR_{i}=1|{\bar{z}}_{t-1},\theta )=\overset{T}{\underset{h=1}{\sum }}\overset{h}{\underset{t=1}{\prod }}\{1-\pi_{it}({\bar{z}}_{t-1},\theta )\}\int_{\Omega_{3}^{T-h}}\int_{\Omega_{2}^{1}}\int_{\Omega_{1}^{h-1}}f_{Y_{1},\text{⋯},Y_{T}}(y_{i1},\text{⋯},y_{iT};z_{i0},\theta )dy_{i1}...dy_{iT}.$$

The mean response probability is then estimated by $\frac{1}{n}\sum_{i=1}^{n}\mathrm{Pr}(BOR_{i}=1|\hat{\theta })$, where $\hat{\theta }$ is the maximum likelihood estimator of θ. As before, we work on the logit scale, use the delta method to obtain the variance and then transform back to construct the confidence interval for the mean response probability.

When confirmation is required, having 2 continued responses of CR/PR before progression, one can replace (7) with (Y_1_…Y_h−1_∈Ω_1_,Y_h_,Y_h+1_∈Ω_2_,Y_h+2_…Y_T_∈Ω_3_) with the sum in (9) going from 1 to T−1.

### 2.7 Testing a difference in probability of response between 2 treatments

The above methods can be applied to single‐arm trials. For a randomised trial where comparing the difference in response probability is of interest, a minor addition is required.

We assume 2n patients are recruited with n patients randomised to each arm. Assumptions for log tumour size ratios and new‐lesion progression remain the same as in Section 2.4. We introduce an arm indicator R to the models, with 0 for control and 1 for experimental arms. The log tumour size ratios are modelled by

$${\left(Y_{i1},Y_{i2},\text{⋯},Y_{iT}\right)}^{\prime }|R,z_{0}∼N({\left(\mu_{i1},\mu_{i2},\text{⋯},\mu_{iT}\right)}^{\prime },\Sigma ),$$

where i indexes the ith patient, μ_i1_=μ_1_+η_1_ R+ω z_i0_,μ_i2_=μ_2_+η_2_ R+ω z_i0_. The new‐lesion progression for T>t is modelled by using logistic models

$$\text{Logit}\{\mathrm{Pr}(D_{it}=1|D_{i1}=\text{⋯}=D_{it-1}=0,z_{i0},\text{⋯},z_{i(t-1)})\}=\alpha_{t}+\beta_{t}R+\gamma_{t}z_{i(t-1)}.$$

The probabilities of new‐lesion progression for control and experimental arms are ${\left[1+\exp\{-(\alpha_{t}+\gamma_{t}z_{i(t-1)})\}\right]}^{-1}$ and ${\left[1+\exp\{-(\alpha_{t}+\beta_{t}+\gamma_{t}z_{i(t-1)})\}\right]}^{-1}$, respectively. Let θ be the vector of parameters with length $\left(T^{2}+\frac{9T}{2}+2\right)$ from the above models. The mean response probability at a fixed time is estimated by

$$\bar{\mathrm{Pr}}(S=1|\hat{\theta },R)=\frac{1}{2n}\overset{2n}{\underset{i=1}{\sum }}\mathrm{Pr}(S_{i}=1|\hat{\theta },R),$$

where $\hat{\theta }$ is the maximum likelihood estimator of θ. We note that patients from both arms are included in the calculation of the probability of response in an arm, as is recommended and justified in Wason and Seaman.^7^ The mean difference in response probability at a fixed time is defined as the difference between mean response probabilities for the 2 arms. It can be written by

$$m_{F}(\theta )=\bar{\mathrm{Pr}}(S=1|R=1,\theta )-\bar{\mathrm{Pr}}(S=1|R=0,\theta ).$$

We obtain the variance of $m_{F}(\hat{\theta })$ by using the delta method and use the Wald test to test whether m_F_(θ) is 0. Similarly, we define the mean difference in response probability for BOR as

$$m_{B}(\theta )=\bar{\mathrm{Pr}}(BOR_{i}=1|R=1,\theta )-\bar{\mathrm{Pr}}(BOR_{i}=1|R=0,\theta ).$$

Both the extended and modified methods can be used as in previous sections. Moreover, we provide a package mAugbin in R including the extended augmented binary method as well as the modified augmented binary method. Changing of integral regions to adapt RECIST or user defined criteria is allowed.

## 3 RESULTS

In this section, we evaluate the performance of the proposed methods in terms of precision and power using simulations and a real data example. We use “Bin” to represent the method that just analyses the response outcomes as binary. For single‐arm trials, the binary method uses the R‐package Hmisc to construct a Wilson interval for binary success (S=1 or B O R=1). For 2‐arm studies, the binary method is a logistic regression model that has parameters for treatment group and baseline tumour size, from which the treatment effect can be tested. The terms “Augbin,” “eAugbin,” and “mAug” refer to methods that use continuous information. They are, respectively, Wason and Seaman's method^7^ at 2 follow‐up times, the extended method for more than 2 follow‐up times, and the modified method for rapid computation. We use fixed time with varying numbers of follow‐up times with c_T_ and c being log(0.7) and −∞ and BOR without confirmation as the endpoints.

### 3.1 Simulation study setup

Following the aforementioned notation, the observed data available for each patient is (F_i_,**z**_i_). The observed data are simulated as follows. First of all, baseline tumour size z_i0_ for patient i is generated from a uniform distribution and log tumour size ratios of T follow‐up time $\left\{y_{it}:t=1,\text{⋯},T\right\}$ are generated from a multivariate normal distribution. Tumour size z_it_ can then be calculated from $z_{it}=e^{y_{it}}z_{i0}$. Next, new‐lesion progression indicators are generated from logistic models with intercept α and tumour size effect γ. A nonzero γ means that probability of new‐lesion progression depends on the tumour size at the previous time point. We define time to new‐lesion progression as the first time when the new‐lesion progression occurs from the logistic models. Finally, tumour size observations of patient i after progression are replaced as missing.

#### 3.1.1 Single‐arm trials assessing response at fixed time

Before generating 5000 replicates, we test the computation time for running one replicate using Augbin/eAugbin. We generated one replicate of 75 patients. Baseline tumour size (Z_0_) is generated from a uniform distribution and log tumour size ratios are generated from a multivariate normal distribution for 2 to 6 follow‐up times. The (α,γ) are set to (−1.5,0) and (−2.5,0.2). The value of α=−1.5 corresponds to an 18% chance of developing new lesions between each visit. The computation time for running 1 replicate using Augbin/eAugbin for 2 to 6 follow‐up times are 0.04,0.65,2.28,3.41, and 4.47 minutes; while mAug at 6 follow‐up times takes 0.09 minutes. We do not consider T>4 because of the length of time need to simulate 5000 replicates for eAugbin. The simulation settings of log tumour size ratios for 2 follow‐up times is a similar formulation to Wason and Seaman,^7^ that is,

$$Z_{0}∼U(0,1),Y_{2}∼N\left[\begin{array}{c} log(0.7)\left(\begin{array}{c} .5\\ 1 \end{array}\right),\left(\begin{array}{cc} .5 & .5\\ .5 & 1 \end{array}\right) \end{array}\right].$$

The settings for T=3 and4 are Z_0_∼U(0,1),

$$Y_{3}∼N\left[\begin{array}{c} log(0.7)\left(\begin{array}{c} .5\\ .75\\ 1 \end{array}\right),\left(\begin{array}{ccc} .5 & .5 & .5\\ .5 & .75 & .75\\ .5 & .75 & 1 \end{array}\right) \end{array}\right],Y_{4}∼N\left[\begin{array}{c} log(0.7)\left(\begin{array}{c} .25\\ .5\\ .75\\ 1 \end{array}\right),\left(\begin{array}{cccc} .25 & .25 & .25 & .25\\ .25 & .5 & .5 & .5\\ .25 & .5 & .75 & .75\\ .25 & .5 & .75 & 1 \end{array}\right) \end{array}\right].$$

Table 1 shows mean estimated response probability and coverage for Bin, Augbin/eAugbin, and mAug for 2, 3, and 4 follow‐up times for 5000 replicates. The columns 10 to 11 show the reduction in 95% confidence interval (CI). They are, respectively, the average of [1− (CI width of Augbin)/(CI width of Bin)] and [1− CI width of mAug)/(CI width of Bin)]. As seen, in all cases, eAugbin and mAug have narrower CIs compared with Bin. For example, mAug reducing the CI width by 14% means that Bin needs an additional 35% sample size to obtain a similar width. The mAug has a similar coverage to Augbin at t=2. For larger t, it appears the mAug method has a better coverage probability (ie, closer to the nominal value) than eAugbin. The reduction in confidence interval width, compared to the binary method, appears to be similar for the 2 methods. Thus for single‐arm trials it appears mAug shows a significant improvement in computational efficiency without notably poorer statistical characteristics compared to eAugbin.

**Table 1:** Mean estimated probability of response and coverage of the modified augmented binary method (mAug) in comparison with using dichotomised continuous method (Bin), augmented binary method (Augbin), and extended augmented binary method (eAugbin) using fixed time with varying numbers of follow‐up times

			Mean of estimated probability			Estimated coverage			Reduction in width of 95% CI (%)	
Scenario				Augbin/			Augbin/		Augbin/	
(α,γ)	Time	True	Bin	eAugbin	mAug	Bin	eAugbin	mAug	eAugbin	mAug
(−1.5,0)	2	0.334	0.333	0.332	0.338	0.957	0.947	0.947	15.68	14.75
(−2.5,0.2)	2	0.293	0.293	0.293	0.286	0.948	0.945	0.941	13.45	11.94
(−1.5,0)	3	0.318	0.316	0.314	0.317	0.953	0.936	0.949	12.53	13.26
(−2.5,0.2)	3	0.450	0.444	0.443	0.443	0.954	0.943	0.948	14.5	15.28
(−1.5,0)	4	0.270	0.268	0.263	0.268	0.949	0.926	0.95	12.67	12.54
(−2.5,0.2)	4	0.429	0.422	0.421	0.421	0.957	0.938	0.943	13.14	14.41

Abbreviation: CI, confidence interval.

#### 3.1.2 Randomised trials using response at fixed time

We consider a two‐arm trial with a control and experimental arm for 2 follow‐up times. Each arm has 75 patients that have been allocated at random. Baseline (Z_0_) is generated from a U(0,1) distribution. The mean log tumour size ratios between each visit are generated from a normal distribution with mean μ and variance $\frac{1}{2}$. We set μ=log(0.7)+δτ+ψ, where δ=1 for control and δ=−1 for experimental arms, 2τ is the difference in the mean log tumour size ratio and ψ reflects the effectiveness of the control treatment. This is a similar formulation as.^7^

**Figure 1:**
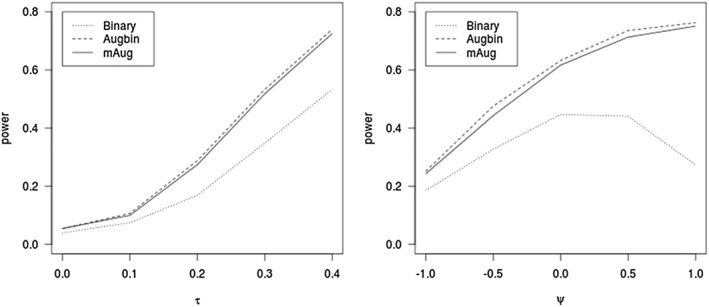
Power of the 3 methods for fixed time as the mean log tumour size ratio (τ) varies and as ψ varies at τ=.35

Figure 1 compares the powers for Bin, eAugbin, and mAug methods for randomised trials. The figure on the right shows the power over treatment effect when τ =.35. As seen, there is a clear power gain when using either mAug or Augbin. mAug performs very closely to Augbin. The empirical Type I error when the difference is 0 for Augbin and mAug are 0.054 and 0.055, respectively.

#### 3.1.3 Noncomparative trials for BOR

Using the binary composite outcome, patients are classified as responders if they have a CR/PR before time F. The computation time for running 1 replicate using Augbin/eAugbin and BOR for 3 to 6 follow‐up times are 0.05,0.09,0.3, and 0.56 minutes; while mAug at 6 follow‐up times takes 0.22 minutes. Again, we use 5000 replicates of 75 patients. Baseline tumour size is generated from a uniform distribution (0, 1). The log tumour size ratios are generated from multivariate normal distribution for 4,5,6, and 7 follow‐up times with $\sigma_{tt}^{2}=1,t=4,5,6,7$. Regardless of the number of visits after baseline, we set the mean log tumour size ratios at the end of the treatment to log(0.7). For example, the case where *T*=4 refers to having 4 visits after baseline and μ being set to 0.25 log (0.7), 0.5 log (0.7), 0.75 log (0.7), log (0.7). For computational reasons, eAugbin was included for up to *T*=6. Table 2 shows the operating characteristics of eAugbin, mAug, and Bin for maximum number of visits varying from 4 to 7. Overall, mAug reduces the average width of the CI by at least 16% compared with Bin. This is equivalent to needing a sample size of around 101 (1.16^2^×75), to obtain a similar average width using Bin. The reduction in width is slightly higher when there is a tumour size effect on new‐lesion progression.

**Table 2:** Mean estimated probability of response and coverage using best observed response without confirmation with Bin, eAugbin, and mAug for maximum number of visits from 4 to 7

(*α*,*γ*)	n	Time	True	Mean of estimated probability			Estimated coverage			Reduction in width of 95% CI(%)	
				Bin	eAugbin	mAug	Bin	eAugbin	mAug	eAugbin	mAug
(−1.5,0)	75	4	0.4	0.4	0.404	0.403	0.959	0.954	0.955	16.5	15.9
(−1.5,0)	75	5	0.391	0.393	0.398	0.396	0.943	0.951	0.952	16.6	15.9
(−1.5,0)	75	6	0.386	0.39	0.395	0.394	0.959	0.954	0.955	16.7	16
(−1.5,0)	150	7	0.382	0.382	—	0.387	0.944	—	0.957	—	16.6
(−2.5,0.2)	75	4	0.46	0.457	0.462	0.461	0.941	0.957	0.957	16.8	17.3
(−2.5,0.2)	75	5	0.452	0.448	0.454	0.452	0.954	0.96	0.96	18.2	17.2
(−2.5,0.2)	75	6	0.446	0.442	0.449	0.447	0.942	0.962	0.961	18.3	17.2
(−2.5,0.2)	150	7	0.441	0.441	—	0.446	0.95	—	0.96	—	18.3

#### 3.1.4 Comparative trials for BOR

**Figure 2:**
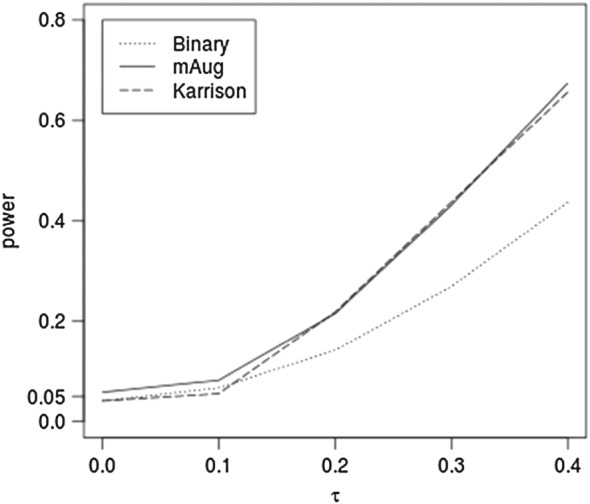
Power of the binary, mAug, and Karrison's methods for best observed response for 4 time points as the mean log tumour size ratio (τ) varies

To illustrate results of the mAug method for a two‐arm trial, we consider the case where each arm has 75 patients and patients are followed for 4 time points. The mean log tumour size ratios for each time point is (log(0.7)+.25δτ), (log(0.7)+.5δτ), (log(0.7)+.75δτ) and (log(0.7)+δτ), where *δ*=1 for control and *δ*=−1 for experimental arms respectively. We also consider the method of Karrison et al.^6^. They proposed to assign the highest observed log tumour ratio (referred to as worst outcome henceforth) for deaths and dropouts, and best possible outcome for complete responders, but do not explicitly say how to deal with BOR. We use worst outcome only if the first non‐baseline observation was a progression and otherwise use the lowest logtumour before progression. Figure 2 compares the powers for Bin, mAug, and Karrison's methods in comparative trials for 4 time points when BOR is used. The empirical type I error when the difference is 0 for Binary, mAug, and Karrison are 0.041, 0.058, and 0.042, respectively. Although there is a slight inflation in type I error rate for mAug, in general, there is a consistent power advantage when using mAug compared to using Bin and the power of mAug is very similar to Karrison's method.

### 3.2 Case study: HORIZON II

The HORIZON II (clinicaltrials.gov identifier: NCT00384176) is a 3‐arm colon cancer trial sponsored by AstraZeneca. Patients initially were randomly assigned 1:1:1 to placebo, cediranib 20 mg once daily, and cediranib 30 mg once daily. Later, subsequent patients were randomly assigned 1:2 to placebo or cediranib 20 mg.^12^ The numbers of patients with baseline record for the 3 arms are 346, 484, and 209. The tumour sizes of patients were measured every 6 weeks up to 24 weeks and then every 12 weeks. Figure S1 in Supporting Information shows a waterfall plot for the individual reduction in tumour size at week 24 from the baseline. There are cases that participants are classified as responders before progression which results in different response estimates between fixed time and BOR.

We used a permutation test to calculate the empirical type I error rate. Data from baseline, 6, 12, 18, and 24 weeks, were used. We simulated 5000 replicates, with the treatment assignment label shuffled randomly in each replicate. For each replicate, we tested the difference in probability of BOR between 2 treatment arms using mAug with 4 follow‐up times. The empirical type I error for no difference between placebo and cediranib 20 mg is 0.0558 and that between placebo and cediranib 30 mg is 0.0518. These are within Monte Carlo standard error of a true type I error of 0.05 (MC error +/− 0.006).

Figure S2 in Supporting Information shows the mean estimated response probability using the 3 methods and fixed time with between 2 and 5 follow‐up times for Placebo, 20 and 30 mg, respectively. The mean estimated response probability decreases as the number of time points increases. Generally, the estimated mean probabilities of response for 3 methods are similar. Figure S3 in Supporting Information shows a residual plot of the fitted multivariate normal model for the 20 mg arm using 3 follow‐up times. The residuals look close to normally distributed, though there is a pattern the variance of the residuals may be decreasing as the fitted values increase. In general, it may be beneficial to apply a transformation such as the Box‐Cox family.

Table 3 reports the width of the 95% CI for each arms probability of response using fixed time. The width corresponds to the length of the vertical lines shown in Figure S2. The 95% CI widths of eAugbin and mAug are considerably narrower than that of Bin. We compared Placebo and cediranib 20 mg as well as Placebo and cediranib 30 mg using mAug BOR and Bin BOR for 4 to 6 time points. Results show that the mAug method gives a considerably smaller 95% CI than the Bin method. The maximum width of the 95% CI for mAug is 0.131 for comparing Placebo with 30 mg, while the width is 0.174 for Bin (See Table S1 in Supporting Information).

**Table 3:** The width of 95% CI for 3 methods using fixed time with between 2 and 5 follow‐up times for individual arm

Method	Time											
	Placebo				Cediranib 20 mg				Cediranib 30 mg			
	2	3	4	5	2	3	4	5	2	3	4	5
Bin	0.113	0.114	0.111	0.105	0.111	0.112	0.111	0.111	0.134	0.133	0.13	0.124
eAugbin	0.073	0.074	0.073	0.07	0.072	0.075	0.074	0.064	0.088	0.088	0.088	0.085
mAug	0.086	0.087	0.087	0.08	0.086	0.088	0.086	0.088	0.105	0.105	0.104	0.096

## 4 DISCUSSION

In this paper, we have considered how the augmented binary method of Wason and Seaman^7^ can be extended to be applicable for a wider range of phase II oncology trials. We have made 3 contributions. The first is to extend the existing method to more than 2 follow‐up times. The second is a modified method that considerably reduces the computational time by making a simplifying assumption about the relationship between new lesions and tumour size change. The third is a mechanism for using both of these methods when the endpoint is based around the best observed RECIST observation before progression, which is a common phase II oncology endpoint.

We have shown that all proposed methods carry the same good properties as the augmented binary method. They provide extra precision, ie, they require a smaller sample size for the same precision (compared to the traditional analysis of analysing response as a binary outcome) in single‐arm trials and are more powerful in comparative trials. We include Karrison's method, which directly tests the continuous tumour change outcome. As found in Wason and Seaman,^7^ in the comparison of using fixed time, Karrison's method performed better than Augbin when the probabilities of new lesions were different between arms and worse when the mean tumour size changes were different, which we expect to be true for BOR as well. We show results comparing the proposed Augbin on BOR and Karrison's method when the probabilities of new lesions are different between arms. We note in this case the Augbin and Karrison's method give similar power. Karrison's method is simpler to implement, but the Augbin estimates a quantity that is more clinically interpretable.

The difference between the modified (mAug) and extended (eAugbin) methods is that the former uses the estimated probability of new‐lesion progression whereas the latter more correctly incorporates variation by averaging all possibilities. Estimation of probabilities using the modified method might be biased if only a few patients remain in a trial at some time point. The mAug has similar properties to eAugbin with respect to precision and power when using BOR.

The extended and modified methods define progression as 20% increase from baseline, whereas RECIST defines progression as 20% increase from the minimum point observed. On the HORIZON II dataset, we examined the number of patients who had their BOR being PR or CR by both of these definitions. The number is the same for both approaches for all number of follow‐up times. This indicates that considering progression as being 20% from baseline does not substantially affect the estimation. However, we should point out that the eAugbin would be able to use the RECIST definition of progression by including a suitable indicator variable in the integrand as well as mAug by changing regions of integration of variables. Details about how to use the true definition can be found in Supporting Information.

All proposed augmented binary methods involve modelling the log tumour size ratio and new‐lesion progression indicators. The new lesion indicator can include other reasons for progression such as unequivocal progression of nontarget lesions. It may also be possible to include a second logistic regression model for the nontarget lesions separately to increase efficiency further. The log tumour size ratio has been shown to be approximately normally distributed in past data in oncology.^13^ Wason and Seaman^7^ show results from the Augbin can be quite sensitive to deviations from the normal assumption. We find that the residuals of the log tumour ratio in our real data application are close to being normal. However, in general, it may be useful to use a transformation to ensure the normality assumption is as close to true as possible. One could also use other models rather than multivariate normal. An alternative approach is joint latent modelling of longitudinal tumour size data and the new‐lesion progression. One can use a random effect model for the repeat tumour size measure and a latent class membership for new‐lesion progression. By membership, we mean a participant has probabilities of belonging to latent classes. Each class refers to the time when new‐lesion progression occurs. Moreover, tumour‐growth progression or new lesions appearing at a time period results in the patient's tumour size measure being missing for all subsequent time periods. Considering this monotone missing pattern in log tumour size, the joint probability of log tumour size can be written as the product of a set of conditional probabilities of current log tumour size ratio given previous data.^14^ Complete responses do cause an issue with the proposed methodology as they mean the log tumour ratio is negative infinity—in practice, we would set complete response to an extreme value of log tumour size ratio. Potentially an alternative model such the censored normal distribution^15^ could be used. Future work is warranted to investigate whether this more complicated methodology is worth applying.

We have only considered ORR in this work. However, the proposed method can be adjusted for using disease control rate where SD is included. The probability of patients being classified as SD can be calculated by adapting the inner integral in equations from log(0.7) to log(1.2). The joint distribution of the probability of being in each ordered category (CR/PR, SD, and PD) could be estimated by suitably extending the delta‐method approach. An increasingly commonly used phase II endpoint is progression‐free survival (PFS). Response‐based endpoints focus on success, and PFS is interested in progression. Further development of the Augbin from response to progression so that it can be applied to improve analyses of PFS is an area of current work.

## 5 SOFTWARE

For estimating the probability of response for fixed time and for BOR, a package mAugbin in R is available at https://sites.google.com/site/jmswason/supplementary-material for the methods proposed in this paper. The package includes both the eAugbin and mAug.

## Supporting information




**Figure S1.** Waterfall plot for individual reduction in tumour size at week 24 from the baseline in comparison to their baseline response for three arms. The colouring shows their best response over the 4 visits. Patients having new‐lesion progression before week 24 are not plotted. As seen, the proportions of responders (CR/PR) at week 24 for three arms are similar. There are cases that participants having classified as responders before progression.
**Figure S2.** Mean estimated response probability of three methods using *fixed time*.The dot indicates the mean estimated response probability and the bar indicates SE.
**Figure S3.** Residual plot of the fitted multivariate normal model for the 20 mg arm using 3‐follow up times. Residuals are obtained from a multivariate normal model with covariates (baseline and time | patients' id). Before model fitting, all negative infinity logtumour size (tumour size zero) is replaced by the minimum of the logtumour size of time 1,2,3, respectively. The colour blue indicates that one of the logtumour size of the 3‐follow up time is negative infinity. The residuals look close to normally distributed, though there is a pattern the variance of the residuals may be decreasing as the fitted values increase. In general it may be beneficial to apply a transformation such as the Box‐Cox family to ensure the normality assumption is as close to true as possible.
**Table S1.** The width of 95% CI of the difference of mean estimated response probability between Placebo and treatments using BOR endpoints at time points 4, 5 and 6. All 95% CIs contain zero.

mAugbin
