# Supplementary material for: Improving phase II oncology trials using best observed RECIST response as an endpoint by modelling continuous tumour measurements
Source: Stat Med. 2017 Aug 28;36(29):4616–26. doi: 10.1002/sim.7453 (PMC5724692; doi:10.1002/sim.7453)
Supplement: Supplementary file 1 — Figure S1. Waterfall plot for individual reduction in tumour size at week 24 from the baseline in comparison to their baseline response for three arms. The colouring shows their best response over the 4 visits. Patients having new‐lesion progression before week 24 are not plotted. As seen, the proportions of responders (CR/PR) at week 24 for three arms are similar. There are cases that participants having classified as responders before progression. Figure S2. Mean estimated response probability of three methods using fixed time.The dot indicates the mean estimated response probability and the bar indicates SE. Figure S3. Residual plot of the fitted multivariate normal model for the 20 mg arm using 3‐follow up times. Residuals are obtained from a multivariate normal model with covariates (baseline and time | patients' id). Before model fitting, all negative infinity logtumour size (tumour size zero) is replaced by the minimum of the logtumour size of time 1,2,3, respectively. The colour blue indicates that one of the logtumour size of the 3‐follow up time is negative infinity. The residuals look close to normally distributed, though there is a pattern the variance of the residuals may be decreasing as the fitted values increase. In general it may be beneficial to apply a transformation such as the Box‐Cox family to ensure the normality assumption is as close to true as possible. Table S1. The width of 95% CI of the difference of mean estimated response probability between Placebo and treatments using BOR endpoints at time points 4, 5 and 6. All 95% CIs contain zero. [file SIM-36-4616-s001.pdf]

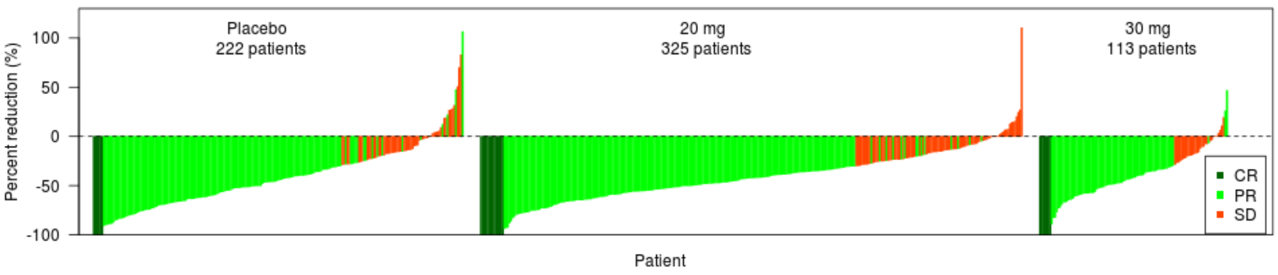

Figure S1. Waterfall plot for individual reduction in tumour size at week 24 from the baseline in comparison to their baseline response for three arms. The colouring shows their best response over the 4 visits. Patients having new-lesion progression before week 24 are not plotted. As seen, the proportions of responders (CR/PR) at week 24 for three arms are similar. There are cases that participants having classified as responders before progression.

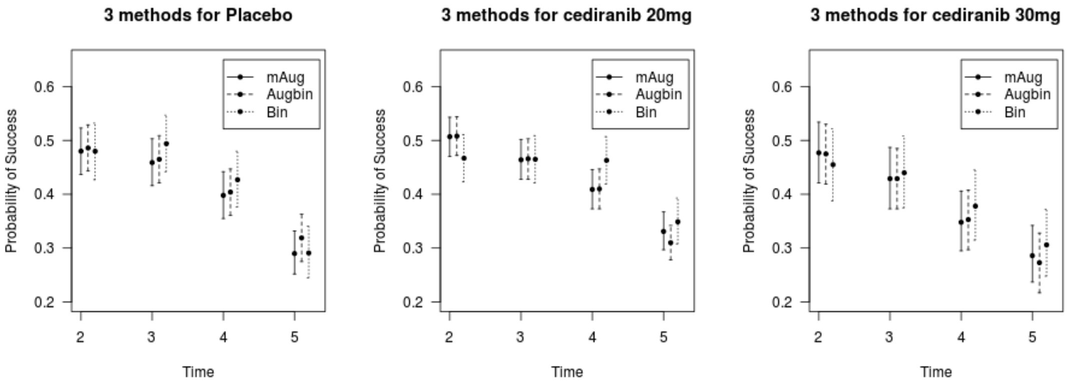

Figure S2. Mean estimated response probability of three methods using *fixed time.*The dot indicates the mean estimated response probability and the bar indicates SE.

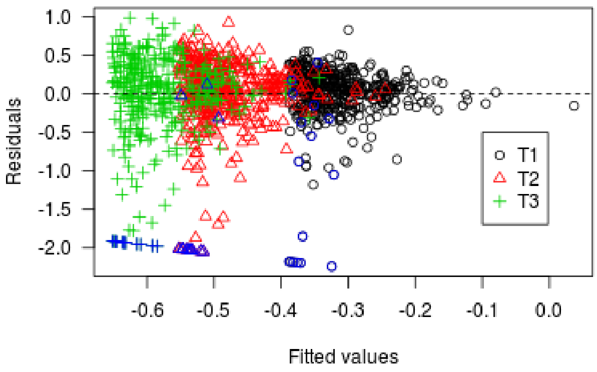

Figure S3. Residual plot of the fitted multivariate normal model for the 20 mg arm using 3-follow up times.

Residuals are obtained from a multivariate normal model with covariates (baseline and time | patients' id). Before model fitting, all negative infinity logtumour size (tumour size zero) is replaced

by the minimum of the logtumour size of time 1,2,3, respectively. The colour blue indicates that one of the logtumour size of the 3-follow up time is negative infinity. The residuals look close to normally distributed, though there is a pattern the variance of the residuals may be decreasing as the fitted values increase. In general it may be beneficial to apply a transformation such as the Box-Cox family to ensure the normality assumption is as close to true as possible.

Table S1. The width of 95% CI of the difference of mean estimated response probability between Placebo and treatments using BOR endpoints at time points 4, 5 and 6. All 95% CIs contain zero.

Method\Time	Placebo v.s. 20 mg			Placebo v.s. 30 mg		
	4	5	6	4	5	6
Bin	0.141	0.14	0.136	0.175	0.175	0.174
mAug	0.105	0.106	0.106	0.13	0.131	0.131

Notation

z_t tumour size at time t, where t=0 is the baseline time.

y_{ab} log tumour size ratio, $y_{ab}=\log(z_a/z_b)$

D_t progression indicators.

S composite response indicator for fixed time

BOR composite response indicator for best observed response

$$S = \begin{cases} 1 \text{ if } D_j\text{=0 for all j=1,…,t, } y_t < \text{log0.7 and no tumour progression before t} \\ 0\text{, otherwise} \end{cases},$$

$$BOR = \begin{cases} 1 \text{ if there exists a t such that } y_t < \text{log0.7, t} \le \text{min (F ,X-1)} \\ 0\text{, otherwise} \end{cases}.$$

A.1 Extended augmented binary method at a fixed time (t=3) with nadir

Assume that log tumour size ratios from baseline follow a multivariate normal distribution, the log tumour size ratios can be modelled by

$$(Y_{10}, Y_{20}, Y_{30})'|\, z_0 \sim N((\mu_1, \mu_2, \mu_3)', \Sigma).$$

The new-lesion progression is modelled by

$$\text{logit}\Pr(D_t = 1 \mid D_1 = \ldots = D_{(t-1)} = 0; z_0, \ldots z_{(t-1)}) = \alpha_t + \gamma_t z_{(t-1)}.$$

The probability of response for patient i at time 3 considering nadir can be written by:

$$\Pr(S_i|\theta) =$$

$$\int_{-\infty}^{\log(1.2)}\int_{-\infty}^{\log(1.2)}\int_{-\infty}^{\log(0.7)} \mathrm{I}[(y_{20}-y_{10}) < \log(1.2)]\ \mathrm{I}[(y_{30}-y_{10}) < \log(1.2)]\ \mathrm{I}[(y_{30}-y_{20}) < \log(1.2)]$$

$$\Pr(D_{i1} = 0|z_0)\Pr(D_{i2} = 0|z_{0,}z_{1,})\Pr(D_{i3} = 0|z_0, z_{1,}, z_{2,}) f_{Y_{10}Y_{20}Y_{30}}(y_{10}y_{20}y_{30})dy_{10}dy_{20}dy_{30},$$

Where I(·) is an indicator function.

A.2 Modified augmented binary method at a fixed time (t=3, t=T) with nadir

Assume that log tumour size ratios from baseline follow a multivariate normal distribution, the log tumour size ratios can be modelled by

$$(Y_{10}, Y_{20}, Y_{30})'|\, z_0 \sim N((\mu_1, \mu_2, \mu_3)', \Sigma).$$

$y_{ab} = log\left(\frac{z_a}{z_b}\right) = logz_a - logz_b + logz_0 + logz_0 = log\left(\frac{z_a}{z_0}\right) - log\left(\frac{z_b}{z_0}\right)$, then

$(Y_{10}, Y_{20}, Y_{30}, Y_{21}, Y_{31}, Y_{32})'| z_0$ can be written as

$(Y_{10}, Y_{20}, Y_{30}, Y_{20}\text{-}Y_{10}, Y_{30}\text{-}Y_{10}, Y_{30}\text{-}Y_{20})'| z_0 \sim N(A\boldsymbol{\mu}^T, A\Sigma A^T)$.

where

$$A = \begin{bmatrix} 1 & 0 & 0 \\ 0 & 1 & 0 \\ 0 & 0 & 1 \\ -1 & 1 & 0 \\ -1 & 0 & 1 \\ 0 & -1 & 1 \end{bmatrix}$$

The probability of response for patient i at time 3 can be written by:

$$\Pr(S_i = 1|\bar{z}_{t-1}, \theta)$$

$$= \prod_{t=1}^{3}\{1 - \pi_{it}(\bar{z}_{t-1}, \theta)\}$$

$$\int_{-\infty}^{\log(1.2)}\int_{-\infty}^{\log(1.2)}\int_{-\infty}^{\log(0.7)}\int_{-\infty}^{\log(1.2)}\int_{-\infty}^{\log(1.2)}\int_{-\infty}^{\log(1.2)} f(y_{10}y_{20}y_{30}y_{21}y_{31}y_{32})dy_{10}dy_{20}dy_{30}dy_{21}dy_{31}dy_{32}$$

The probability of response for patient i at time T can be written by:

$$\Pr(S_i = 1|\bar{z}_{t-1}, \theta) = \sum_{t=1}^{T}\{1 - \pi_{it}(\bar{z}_{t-1}, \theta)\}\int_{\Omega^{T-1}}\int_{-\infty}^{\log(0.7)}\int_{\Omega^k} f(\mathbf{y})\mathrm{d}\mathbf{y},$$

where Ω=(-∞, log(1.2)), $\mathbf{y}=(y_{10}, y_{20}, \ldots y_{T0}, y_{21}, \ldots, y_{T(T-1)})$, $k = \sum_{j=2}^{T}\sum_{i=1}^{j}(i-1)$.

A.2 Augmented binary method using best observed response with nadir

Following the main paper, the probability of being classified CR/PR for the first time at time *h* before progression from baseline is

$$\Pr\left(Y_{10}, \ldots, Y_{(h-1)0} \in \Omega_1, Y_h \in \Omega_2, Y_{(h+1)0}, \ldots, Y_{T0} \in \Omega_3\right) = \int_{\Omega_3^{T-h}}\int_{\Omega_2}\int_{\Omega_1^{h-1}} f(y_{10}, \ldots, y_{T0}; \theta)\, dy_{10} \ldots dy_{T0},$$

where $\boldsymbol{\Omega}$ is the probability domain for the set variables. Intuitively, Ω_1 = (log(0.7), log(1.2)) designates the patient being classified as stable disease and Ω_2 designates the patient being classified as a responder. Response after time h is irrelevant, which is represented by Ω_3= (-∞, ∞). Considering nadir, the probability of being classified CR/PR for the first time at time *h* before tumour progression is

$\Pr\left(Y_{10}, \ldots, Y_{(h-1)0} \in \Omega_1, Y_{ab} \in \Omega_1, a = 1 \ldots h, b = 1 \ldots (a-1), Y_h \in \Omega_2, Y_{ab} \in \Omega_3, a = (h+1) \ldots T, b = 1 \ldots (a-1)\right)$

$$= \int_{\Omega_3^u} \int_{\Omega_2} \int_{\Omega_1^r} f(\boldsymbol{y}; \theta) \mathrm{d}\boldsymbol{y}$$

where $\mathbf{y}=(y_{10}, y_{20}, \ldots y_{T0}, y_{21}, \ldots, y_{T(T-1)})$, $r = (h-1) + \sum_{j=2}^{h} \sum_{i=1}^{j} (i-1)$,

$u = (T-h) + \sum_{j=(h+1)}^{T} \sum_{i=1}^{j} (i-1)$

The probability of best observed response for patient i is

$\Pr(BOR_i = 1 | \bar{z}_{t-1}, \theta) = \sum_{h=1}^{T} \prod_{t=1}^{h} \{1 - \pi_{it}(\bar{z}_{t-1}, \theta)\} \int_{\Omega_3^u} \int_{\Omega_2} \int_{\Omega_1^r} f(\boldsymbol{y}; \theta) \mathrm{d}\boldsymbol{y}$.

A.3 Sequential missingness at random

Definition of sequential missingness at random is that conditionally on past history, the full-data response vector, drop-out at time t does not depend on current or future response data.

The full-data log tumour size vector is $Y_i=(Y_{i1},\ldots,Y_{iT})^T$. Let R be indicator variables $R_i=(R_{i1},\ldots,R_{iT})^T$, where R_{it} =1if Y_{it} is observed and =0 otherwise. Tumour progression occurring at time t means $\{Y_{it}=\log(z_{it}/ \min(z_{i0},\ldots z_{i(t-1)}) > \log(1.2)$ and $R_{i0} =\ldots= R_{i(t-1)}=1\}$, and R_{it} =1as Y_{it} is observed.

$\Pr(R_{i(t+1)}=0| R_{it}=1, Y_{i1},\ldots,Y_{iT}) = \Pr(R_{i(t+1)}=0| R_{it}=1, z_{i0},\ldots,z_{iT}) = \Pr(R_{i(t+1)}=0| R_{it}=1, z_{i0},\ldots,z_{it})$, that is, the missing at time (t+1) does not depend on current or future tumour size.

We assume that the probability of new-lesion progression depends only on the observed tumour size at the previous visit, and model the new lesion progression by

$$\text{Logit}\{\Pr(D_{it} = 1|D_{i1} =\ldots = D_{i(t-1)} = 0; z_{i0},\ldots, z_{i(t-1)})\} = \alpha_t + \gamma_t z_{i(t-1)},$$

hence,

$\Pr(D_{it} = 1|D_{i1} =\ldots = D_{i(t-1)} = 0; z_{i0},\ldots, z_{i(t-1)})=\text{expit}(\alpha_t + \gamma_t z_{i(t-1)})$.

$\Pr(\mathrm{D_{it}} = 0 | \mathrm{z_{i0}}, \ldots, \mathrm{z_{iT}}) = \Pr(\mathrm{D_{it}} = \mathrm{D_{i(t-1)}} = \cdots = \mathrm{D_{i0}} = 0 | \mathrm{z_{i0}}, \ldots, \mathrm{z_{iT}})$

$$= \prod_{j=1}^{t} \Pr\left(\mathrm{D_{ij}} = 0 \middle| \mathrm{D_{i(j-1)}} = 0, \mathrm{z_{i0}}, \ldots, \mathrm{z_{i(j-1)}}\right) \forall\, \mathrm{t} > \mathrm{j}$$
