# Supplementary material for: Improving phase II oncology trials using best observed RECIST response as an endpoint by modelling continuous tumour measurements
Source: Stat Med. 2017 Aug 28;36(29):4616–26. doi: 10.1002/sim.7453 (PMC5724692; doi:10.1002/sim.7453)
Supplement: Supplementary file 2 — mAugbin [file SIM-36-4616-s002.gz › mAugbin/html/00Index.html]

R: The Augmented Binary Method

# The Augmented Binary Method

---

## Documentation for package ‘mAugbin’ version 1.0

- DESCRIPTION file.

## Help Pages

|  |  |
| --- | --- |
| Augbin | Augmented binary method for single arm using fixed time |
| Augbin.2arm | Augmented binary method for two arms using fixed time |
| BORAugbin | Augmented binary method for single arm using best observed response (BOR) without confirmation |
| BORAugbin.2arm | Augmented binary method for two arms using best observed response (BOR) |
| BORmAugbin.1arm | Modified augmented binary method for single arm using best observed response (BOR) without confirmation |
| BORmAugbin.2arm | Modified augmented binary method for two arms using best observed response (BOR) |
| failure | failure. |
| mAugbin.1arm | Modified augmented binary method for single arm using fixed time |
| mAugbin.2arm | Modified augmented binary method for two arms using fixed time |
| tumoursize | tumoursize. |
